# Acute bladder decentralization in hound dogs: Preliminary results of effects on hypogastric nerve electroneurograms and detrusor pressure responses to spinal root and hypogastric nerve stimulation

**DOI:** 10.1371/journal.pone.0215036

**Published:** 2019-04-10

**Authors:** Ekta Tiwari, Mary F. Barbe, Michel A. Lemay, Danielle M. Salvadeo, Matthew W. Wood, Michael Mazzei, Luke V. Musser, Zdenka J. Delalic, Alan S. Braverman, Michael R. Ruggieri

**Affiliations:** 1 Department of Electrical and Computer Engineering, College of Engineering, Temple University, Philadelphia, Pennsylvania, United States of America; 2 Department of Anatomy and Cell Biology, Lewis Katz School of Medicine, Temple University, Philadelphia, Pennsylvania, United States of America; 3 Department of Bioengineering, College of Engineering, Temple University, Philadelphia, Pennsylvania, United States of America; 4 Department of Surgery, Temple University Hospital, Temple University, Philadelphia, Pennsylvania, United States of America; University Medical Center Utrecht, NETHERLANDS

## Abstract

**Objective:**

We aimed to refine electroneurogram techniques for monitoring hypogastric nerve activity during bladder filling, and then examined nerve activity in normal intact versus acutely decentralized bladders.

**Methods:**

Effects of electrical stimulation of hypogastric nerves or lumbar ventral roots on detrusor pressure were examined, as were effects of isoflurane versus propofol anesthetics on hypogastric nerve stimulation evoked pressure. Hypogastric nerve activity was then recorded using custom-made bipolar cuff electrodes during bladder filling before and after its transection between the spinal cord and electrode to eliminate efferent nerve signals.

**Results:**

Electrical stimulation of hypogastric nerves evoked low amplitude detrusor pressures that did not differ between the two anesthetics. Upper lumbar (L2) ventral root stimulation evoked detrusor pressures were suppressed, yet not eliminated, after transection of hypogastric nerves and all spinal roots below L5. Afferent and efferent hypogastric nerve activity did not change with bladder filling in neuronally intact bladders yet decreased in decentralized bladders. No change in afferent activity was observed during bladder filling in either intact or decentralized bladders.

**Conclusions:**

These findings indicate that a more complete decentralized bladder model should include transection of lumbosacral spinal roots innervating the bladder as well as hypogastric nerves. These refined electroneurogram recording methods may be suitable for evaluating the effectiveness of nerve transfer surgeries for bladder reinnervation by monitoring sensory activity in the transferred nerve.

## Introduction

Spinal cord injury (SCI) disturbs normal bladder function, reduces quality of life, and can limit social contacts [1]. Restoration of bladder function is consistently identified as a top recovery priority for people with SCI [2, 3]. Understanding afferent contributions to normal bladder function is an area of rapidly growing interest for the treatment of lower urinary tract dysfunction [4–6].

Electroneurogram (ENG) recording is deemed the most suitable method for monitoring afferent activity [7]. In rats, increased afferent activity was recorded from postganglionic branches of the pelvic nerve (located between the major pelvic ganglion and the bladder) [8]. In a porcine model, increased afferent activity was recorded from the right pelvic nerve and right Sacral (S) 2 and S3 roots during bladder filling [9]. In felines, activity of the S1 root during slow bladder filling resulted in a small (<5%) increase in nerve activity with increased bladder pressure [10]. In 6 human patients with SCI, afferent activity recorded from the S3 root extradurally increased with successive bladder fillings [11]. However, there were difficulties in obtaining high signal to noise ratios (SNR) in recorded nerve signals and variability in recording results between subjects.

Successful recording of axonal firing activity in bladder nerves is challenging due to their small diameters, difficulties in accessing appropriate implantation sites for placement of recording electrodes, and artifacts induced by bladder movement during filling and emptying. Hook and cuff electrodes are the most popular designs for ENG recordings [12–14]. Hook electrodes are easier to use for small diameter nerves in regions with limited space as well as for single fiber recording. However, it is difficult to eliminate unwanted signals from surrounding tissues such as skeletal muscle. Use of cuff electrodes can reduce unwanted signals with consideration of design factors, e.g., electrode dimensions, configurations, closure methods and inclusion of insulated silicon sheaths around electrode contacts [14–17]. There is a need for improved recording techniques and high quality signal processing methods for the detection of small amplitude neural signals in bladder nerves.

In normal intact dogs, cell bodies of most sensory axons innervating the bladder are located in dorsal root ganglia (DRG) at lumbar (L) 7 through sacral (S) 3 levels. Axons of these DRG cells travel to the bladder via many routes including with pelvic nerves, sacral branches of the lumbosacral plexus and associated arteries. Other sensory axons to the bladder originate in DRG located at thoracic (T) through L2 and travel with hypogastric nerves to the bladder. Motor axons innervate the bladder through sympathetic and parasympathetic pathways, as well as a few direct projections from the spinal cord (specifically from sacral ventral horn medial lamina regions) [18–20].

This study is part of a larger project examining whether surgical rerouting of lumbar-originating nerves to pelvic nerves allows restoration of bladder function, including continence (storage) and emptying (micturition) functions, in lower motor neuron lesioned bladders. Bladder reinnervation is performed after long-term decentralization. Therefore, defining the best bladder decentralization method is critical to these studies, as is clear assessment of sensory and motor innervation in decentralized versus normal intact bladders. Also, in order to explore if sensory activity in the transferred nerves is induced by bladder filling, refined methods of ENG recordings are needed. Here, we first sought: 1) to refine ENG recording methods, and 2) to apply them in step-by-step surgical electrophysiological experiments, focusing on monitoring ENG recording of hypogastric nerve activity during bladder storage in dogs with intact versus acutely decentralized bladders.

## Materials and methods

### Animals

This study was approved by the Institutional Animal Care and Use Committee of Temple University, and was compliant with National Institutes of Health, United States Department of Agriculture and American Association for Assessment of Laboratory Animal Care guidelines. Thirty-six female mixed-breed hounds dogs, weighing 20–25 Kg and between 6–8 months of age, were used from Covance Research Products Inc. (Cumberland, VA) or Marshall BioResources (North Rose, NY). Because this study is part of a larger study of nerve transfer for restoration of bladder, urethral and anal sphincter function, the numbers of animals per group were decided based on a power analysis of the estimated proportion of successful responses to the reinnervation surgery and not any of the outcome measures included in this manuscript.

### Technical validation

#### Electrode design

Tripolar and bipolar cuff electrodes were designed with different diameters, modified from previous designs [17]. For smaller diameter electrodes, we designed contacts of 1mm widths without flaps (rather than past used 1 mm contact widths with 1 mm flaps on each side). For smaller contacts, instead of spot welding, we soldered the lead wires onto electrode contact backs using minimal voltage to avoid damaging the platinum sheet. Functionality of the electrodes were then tested using traditional continuity, bubble and impedance tests.

#### Noise calibration in saline using different amplifiers

We performed step-by-step testing of each block of the design to calibrate noise in saline (S1 Fig.). Electrode contacts were connected to the differential amplifier and analog-to-digital converter (A/D converter) that were then connected to a computer. Testing was performed with both an AC power line operated amplifier (Model 1700 with an extended head stage, A-M System, Carlsborg, WA) and a battery-operated low noise voltage preamplifier (Model SR560, Stanford Research Systems, Sunnyvale, CA) to measure noise in saline without any external current. A ground connection used to reduce interference from the surrounding environment was placed in the saline close to the contacts. All recordings were performed at a gain of 10k, band pass filtered between 500 Hz-3 kHz and sampled at 20 kHz using PowerLab A/D converter (ADInstruments Inc, Colorado Springs, CO) and displayed using LabChart software.

### In-vivo studies

#### Surgical preparation

**Fig 1:**
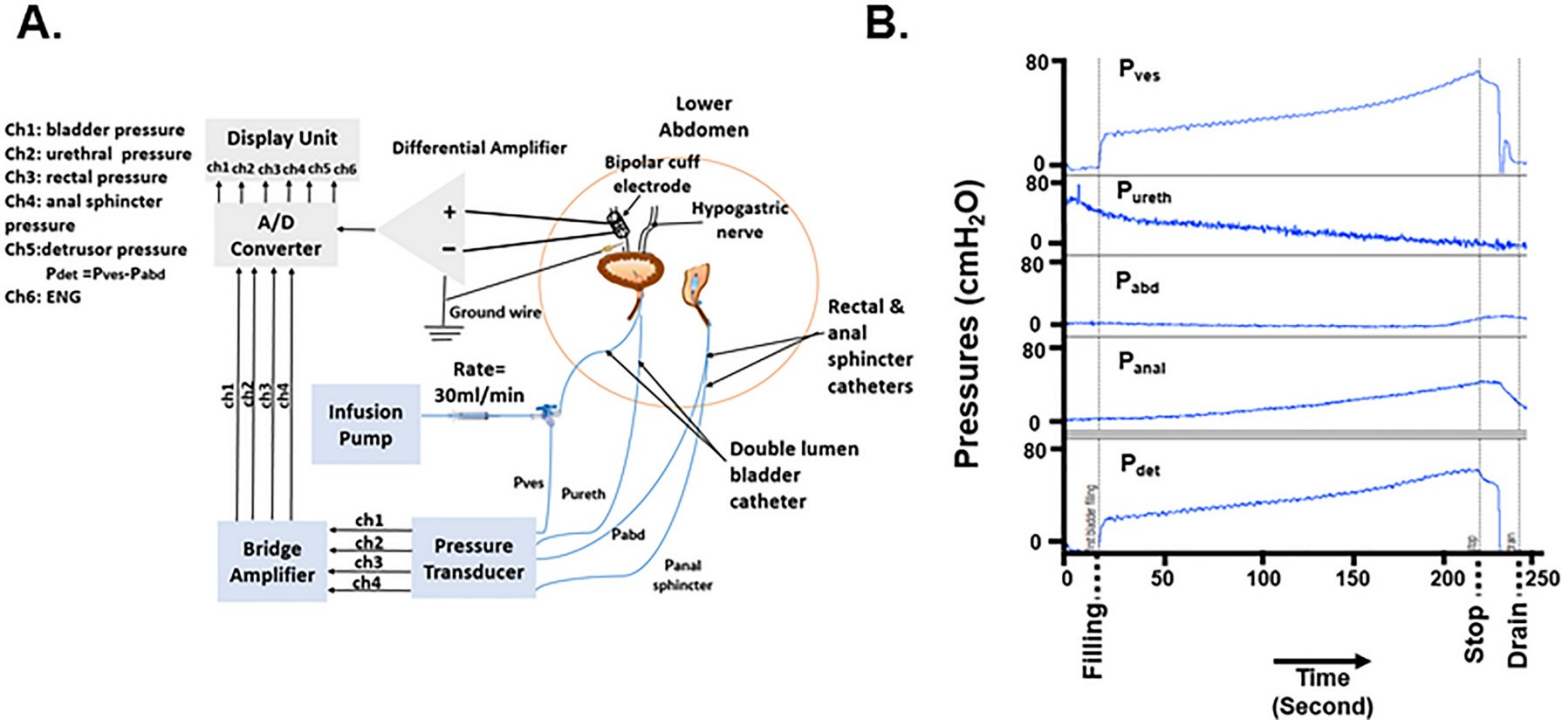
*In-vivo* experimental setup and filling cystometrograms. (A) In-vivo experimental setup to measure pressures and to perform hypogastric nerve stimulation and recording in intra-abdominal region. (B) Example of filling cystometrogram pressures curves. Pressure recordings were performed during bladder filling with a saline infusion at a rate of 30 mL/min. Pves: Intravesical pressure; Pureth: Urethral pressure; Pabd: Abdomen pressure; Panal sphincter: Anal sphincter pressure; Pdet: Detrusor pressure. ENG: Electroneurogram.

Animals were acclimated to the animal facility for at least 7 days prior to the initial surgery. One day prior to surgery, surgery prophylactic antibiotics (2.5-5mg/kg Enrofloxacin P.O.) were started. On the morning of surgery, preanesthetic analgesics were administered (2mg/kg Ketofen IM). Surgical experimental setup and urodynamic procedure are shown in Fig 1A and 1B. Dogs were sedated with 6 mg/kg of propofol, i.v., and then anesthesia maintained using isoflurane at 2–3% maximum alveolar concentration. The bladder was catheterized through the urethra with a dual balloon Foley catheter connected to external pressure transducers for recording bladder (Pves) and ureteral sphincter pressures (Pureth). The bladder pressure (also termed as intravesical pressure) line was connected with a “T” connector to an infusion pump for bladder filling. Balloon-tipped catheters connected to pressure transducers were inserted into the rectum as a surrogate for abdominal pressure (Pabd) and anal sphincter (Panal) for detection of its contractions. Detrusor pressure (Pdet) was calculated by subtracting the abdominal pressure to intravesical pressure (Fig 1A). Pressures were continuously monitored throughout surgeries, as were vital signs, including heart rate, respiration rate, blood oxygen level and body temperature and documented every 15 minutes.

Three successive filling cystometrograms were performed to determine bladder capacity (defined as the infused volume inducing a marked increase in the slope of the volume-pressure curve) with a saline infusion rate of 30 mL/min (Fig 1B). Pressure transducers were attached to bridge amplifiers and analog to digital converter. After the procedure, as the animal emerged from the anesthesia, acepromazine (1 mg/kg SC) was administered to allow the animal to rest comfortably during the immediate post-operative period. The animal was placed in a heated, ventilated recovery cage for 24 hours after surgery. Post-operatively all dogs were given prophylactic antibiotics (2.5mg/kg Enrofloxacin IM BID for 3–4 days then 2.5-5mg/kg Enrofloxacin PO for an additional 6–7 days) and analgesia (2mg/kg Ketofen IM BID for 3–5 days and 0.0075 mg /kg buprenorphine for 24 hours) after the procedure. In acute surgical procedure animals were euthanized while under isoflurane anesthesia by a terminal dose of Euthasol (pentobarbital sodium 86 mg/kg and phenytoin sodium 11 mg/kg IV).

#### Hypogastric nerve stimulation

Hypogastric nerves were stimulated in 36 animals as shown in S2 Fig, with the bladder filled to approximately 50% capacity. First, under isoflurane anesthesia, in 21 animals with intact bladder innervation, the abdomen was entered using a midline incision and hypogastric nerves and caudal mesenteric ganglia were located. Nerves were stimulated near the ganglion (3-10mA, 20Hz, 0.2 msec) using hand-held monopolar or bipolar electrodes from Medtronic Xomed Inc., Jacksonville, FL and maximum detrusor pressures (P_det_) were recorded resultant from multiple stimulations. When distinct nerves were not present, stimulation was applied to the hypogastric plexus. Five animals were then switched to propofol (0.3–0.5 mg/kg/min, iv.) for up to 20 minutes after turning off the isoflurane. The hypogastric nerve stimulation was then repeated.

In another 15 animals, acute extradural bladder decentralization was performed, modified from previously described methods [21]. Briefly, the lower spinal cord and spinal roots were exposed. Hypogastric nerves were left intact. In 3 animals, all dorsal and ventral roots caudal to L7 were transected bilaterally. Another 2 animals underwent bilateral transection of L7 dorsal roots, while 7 underwent additional bilateral transection of L6 & L7 dorsal roots. Because no differences were found between these two latter groups, they were combined for analyses. After root transections, hypogastric nerve stimulation evoked pressures (P_det_) were recorded. In the remaining 3 animals, all spinal roots below L5 were transected, before hypogastric nerve stimulation evoked pressures (P_det_) were recorded (S2 Fig).

#### Lumbosacral spinal cord/roots stimulation

We systematically stimulated L1-S3 spinal segments or roots (mostly ventral roots) using hand-held monopolar or bipolar electrodes in 8 animals with intact bladders while recording pressure (P_det_): 5 sham-operated controls at 6 months after sham spinal root transection, and 3 unoperated controls (S2 Fig). Two of 5 sham-operated controls were excluded from analysis. In one, S2 root stimulation evoked pressures (P_det_) up to 250 cmH_2_O; this was considered as an outlier. In the other, a very significant amount of scar tissue was present on the left L7 dorsal root and S1-S2 cord segments. In the 3 unoperated controls, we next bilaterally transected the L6 & L7 dorsal roots and all roots caudal to L7 (hypogastric nerve left intact) before stimulating the L2 ventral root while recording pressure (P_det_). In these 3 animals, we then bilaterally transected all roots below L5, as well as hypogastric nerves, before stimulating the L2 roots (S2 Fig).

#### Hypogastric nerve recording

**Fig 2:**
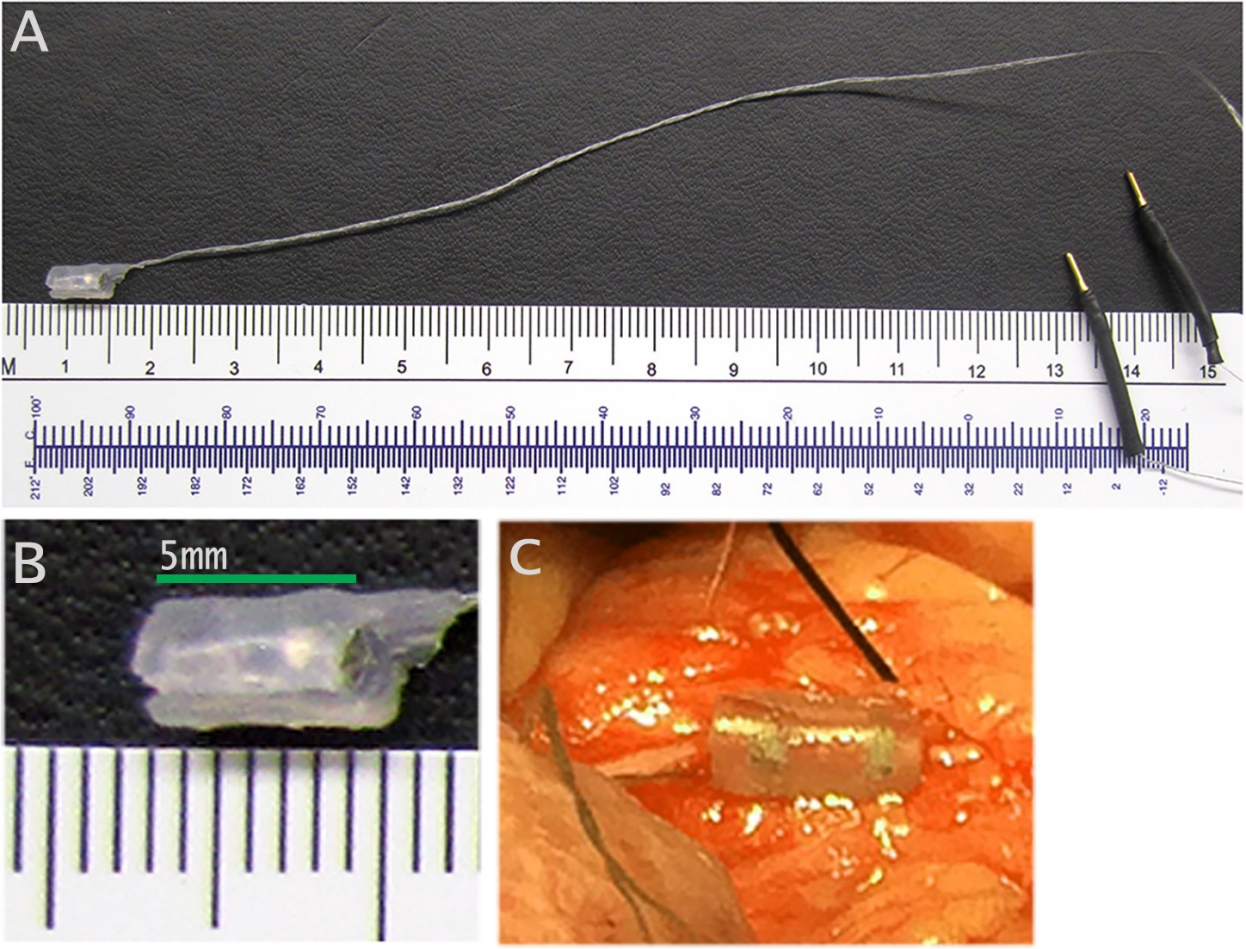
Bipolar cuff electrode used. (A) Final version of electrode. (B) Side view of electrode: two platinum contacts are (25μm thick) separated by 1.5mm; total length: 5.0 mm; inner diameter: 0.8–1.2 mm. (C) Electrode wrapped around a hypogastric nerve.

Following hypogastric nerve stimulation, a total of 31 recordings in 15 dogs were performed from hypogastric nerves during bladder filling using battery operated low noise voltage preamplifier (S2 Fig). All nerve activity was recorded using the final version of our custom-made bipolar cuff electrodes (Fig 2A and 2B). The cuff electrode was wrapped around the hypogastric nerve in the mid abdominal region near the caudal mesenteric ganglion (Fig 2C). The cuff closure was performed using 4.0 silk suture (not shown). We calibrated baseline noise in the abdominal cavity prior to performing recordings. Impedance was measured between the two electrode contacts to establish the integrity of the electrode-nerve connection prior to beginning recordings between 3–5 kΩ. This range was similar to the typical impedance range (2–5 kΩ) measured in past study [22].

Sixteen nerve recordings were performed from 10 animals with intact bladder innervation during two successive fillings (10 during the 1st filling and 6 during the 2nd filling) using a low noise battery operated amplifier (S2 Fig). The bladder was drained completely pre- and post-filling. Five afferent nerve recordings were performed in 5 animals after unilateral nerve transection between the caudal mesenteric ganglion and recording electrode in order to eliminate efferent signals. In the other 5 animals, a total 5 recordings were performed after acute bilateral transection of L6 & L7 dorsal roots and all caudal roots. In these same 5 animals, 4 recordings were performed during a 2nd filling after hypogastric nerve transection (S2 Fig). In 2 of these 5 acutely decentralized animals, intra-abdominal recordings were performed with the recording electrode in the abdomen without any nerve-electrode contact in order to determine whether any mechanical bladder pressure (P_ves_) during filling induced changes in nerve activity.

#### Hypogastric nerve collection and staining

To verify that we were stimulating or recording from hypogastric nerve specifically, 5 to 10 cm long nerve segments from 5 animals were fixed, cryoprotected in 30% sucrose, frozen and cryosectioned longitudinally into 14 micrometer (μm) thick sections. Sections were mounted onto coated slides, dried overnight and immunostained as previously described [23] with an antibody specific for tyrosine hydroxylase (Millipore, #: JC1693653, 1:100 dilution in PBS) visualized with a secondary antibody conjugated to Cyanine Cy3, diluted 1:100 in PBS (Jackson ImmunoResearch, #111-165-144). Negative control staining was performed by omitting the primary antibody.

### Statistical analysis

The mean baseline pressure (P_det_) and peak pressure (P_det_) were calculated by taking 10 data points within 1 second time window. Maximum pressure (P_det_) was determined by subtracting the mean baseline pressure (P_det_) from the mean peak pressure (P_det_) (S3A Fig). Real time processing was performed on recorded ENG signals (S3B Fig). The filtered signals were time-averaged (root mean square) within a 10 second moving window. Bladder pressures (P_ves_) and amplitudes of hypogastric nerve activity were calculated at 0% (same as calibrated baseline noise amplitude in abdominal cavity), 50%, 75% and 100% of bladder capacity. Because the Bartlett test revealed statistically significant differences in variances between groups, nonparametric statistical analysis was used. Median ± interquartile range are reported throughout. The Mann-Whitney U test was performed to evaluate differences between groups. For all statistical evaluations, a p ≤ 0.05 was considered statistically significant.

## Results

### Technical validation

These results for refinements in the methods for ENG recordings are included in the supplemental material.

### Results from in-vivo studies

#### Maximum detrusor pressure obtained during hypogastric nerve stimulation

**Fig 3:**
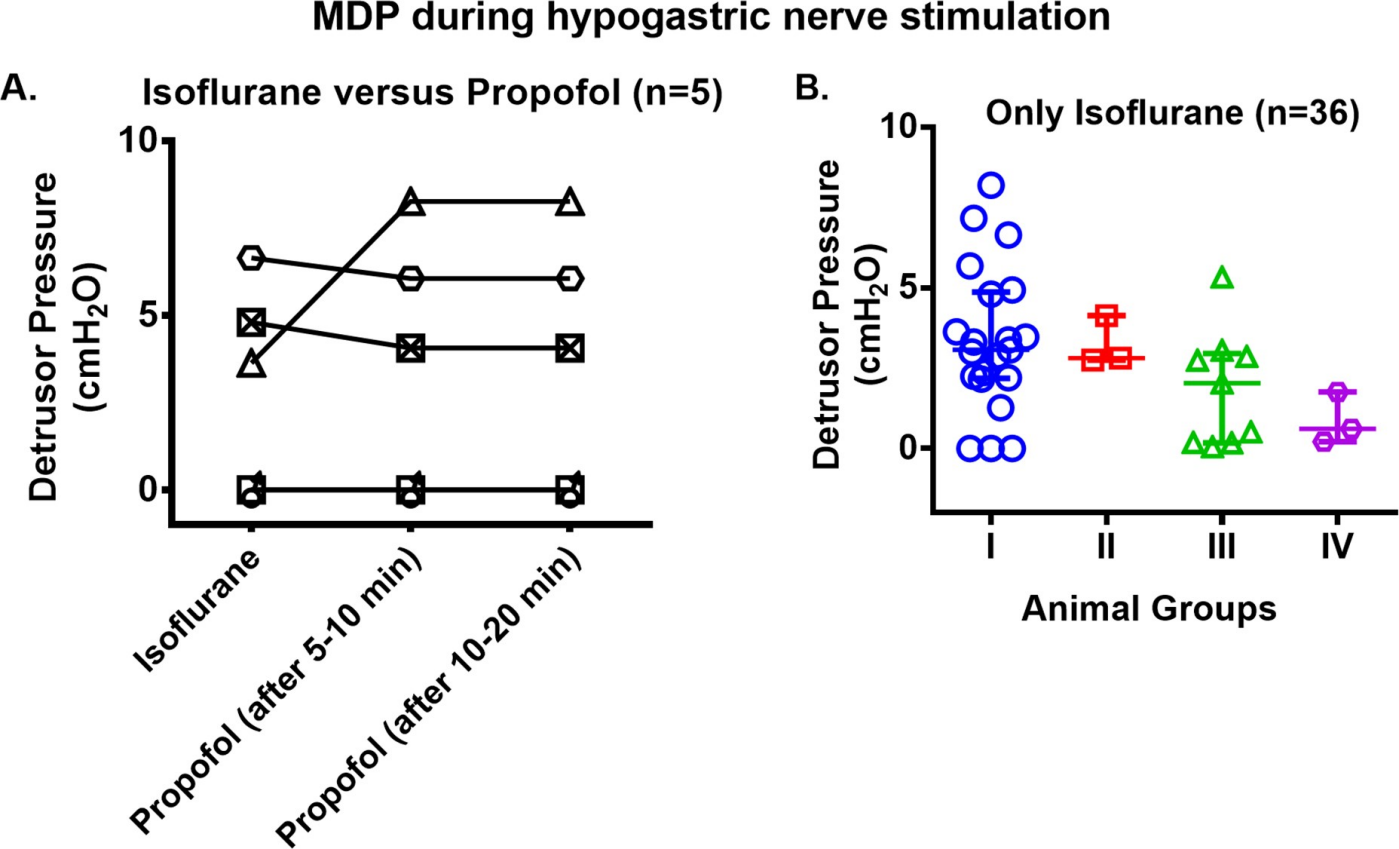
Maximum detrusor pressure (MDP) obtained during hypogastric nerve stimulation. (A) Under isoflurane and propofol anesthesia; and (B) under only isoflurane anesthesia. Groups: I) intact bladder innervation (n = 21); II) bilateral transection of all roots caudal to L7 (n = 3); III) bilateral transection of L7 dorsal roots or both L6 & L7 dorsal roots and all roots caudal to L7 (n = 9) and IV) bilateral transection of all spinal roots below L5 (n = 3). Symbol representation: (A) Each symbol represents individual animal under different anesthesia; (B) Group I: blue circles; Group II: red squares; Group III: green triangles; Group IV: purple hexagons. Abbreviations: L–Lumbar.

We first determined if the anesthetic type altered evoked pressure (P_det_) since some prior studies suggest that isoflurane may reduce sensory or motor evoked potentials [24, 25] and that propofol may reduce motor evoked potentials [26]. Five animals with intact bladder innervation were tested using isoflurane, and then propofol. After switching from isoflurane to propofol (Fig 3A), one animal showed increased pressure (P_det_), while the remaining showed either no change (n = 2) or only a very slight decrease (n = 2) in response to hypogastric nerve stimulation. There was no significant difference between the two anesthetics in evoked pressure (P_det_). Thus, the remaining studies were performed using isoflurane, our veterinarian preferred anesthetic.

We next tested the effects of acute decentralization strategies on hypogastric nerve stimulation evoked pressures (P_det_) (Fig 3B). Nerve stimulation evoked pressure of 3.1 ± 2.7 in intact bladders (Group I); 2.8 ± 1.4 after bilateral transection of all roots caudal to L7 (Group II); 2.0 ± 2.8 after either bilateral transection of L7 dorsal roots and all caudal roots or L6 & L7 dorsal roots and all caudal roots (Group III); and 0.6 ± 1.6 after bilateral transection of all spinal roots below L5 (Group IV). There was no statistically significant difference between the groups (Group II, III & IV), compared to intact bladders (Group I).

#### Maximum pressure obtained during spinal cord/roots stimulation

**Fig 4:**
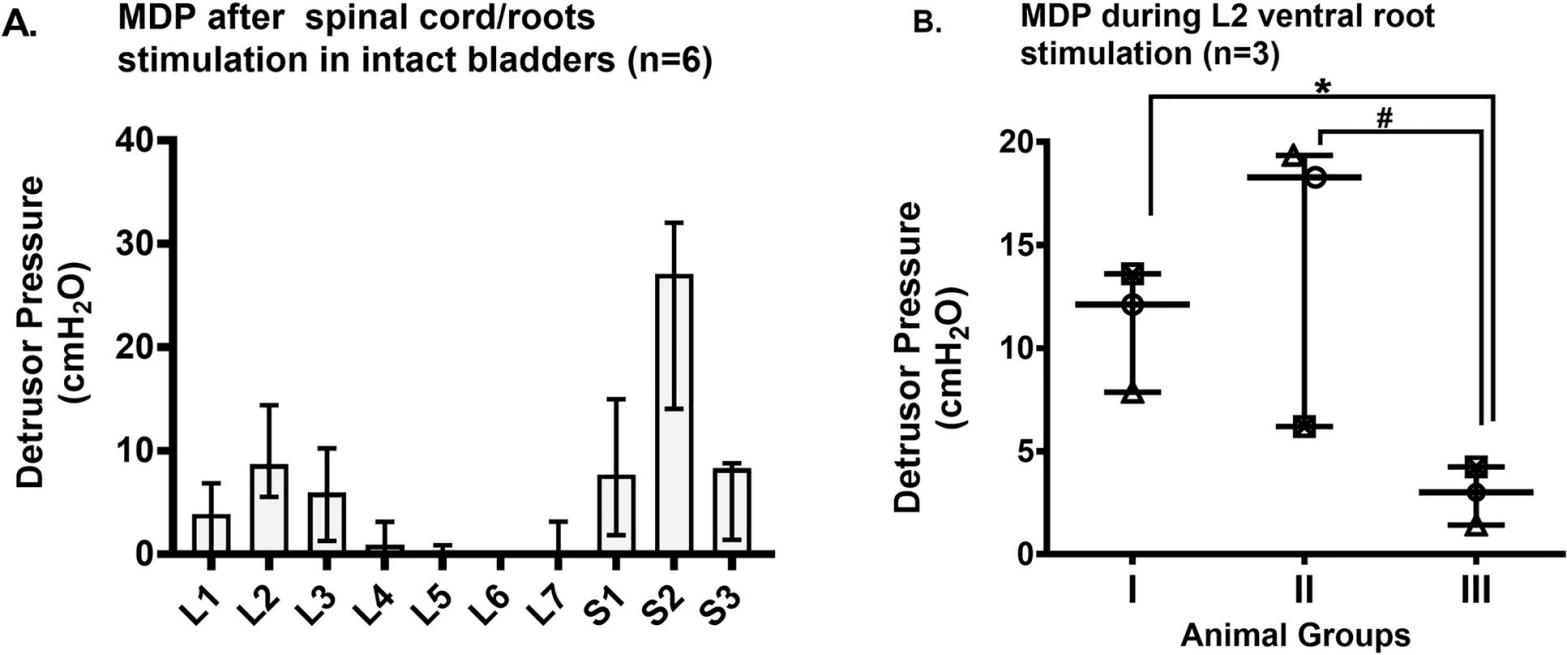
Maximum detrusor pressure (MDP) obtained during spinal cord/roots stimulation. A) Maximum detrusor pressure (MDP) obtained during lumbosacral (L1-S3) spinal segment/root stimulation (n = 6). B) MDP obtained during unilateral L2 ventral root stimulation under isoflurane anesthesia (n = 3). Groups: I) intact bladder innervation; II) bilateral transection of L6 & L7 dorsal roots and all roots caudal to L7, and intact hypogastric nerves; III) bilateral transection of all spinal roots below L5 and bilateral transection of hypogastric nerve. *p = 0.05 (group I & III), #p = 0.05 (group II & III). Abbreviations: L–Lumbar; S- Sacral.

Spinal cord/roots (S1-S3) stimulations showed that S2 stimulation evoked the highest pressure (P_det_) in intact bladders (n = 6; Fig 4A). L1-L7 spinal cord/roots stimulations showed that L2 stimulation also evoked high pressures (P_det_) in intact bladders. The L2 ventral root was stimulated in 3 additional animals undergoing step-wise root/nerve transections in order to observe effects of each surgical step on evoked pressure (P_det_) (Fig 4B). No significant changes were observed with L2 ventral root stimulation in animals with intact hypogastric nerves and after bilateral transection of the dorsal roots of L6 & L7 and all roots caudal to L7 (Group II) versus animals with intact bladders (Group I). However, we observed a statistically significant decrease in pressure (P_det_) after bilateral transection of all spinal roots caudal to L5 and bilateral transection of hypogastric nerves (Group III), compared to intact bladders (p = 0.05), and also compared to decentralized bladders that included bilateral transection of L6 & L7 dorsal roots and all roots caudal to L7 and intact hypogastric nerve (p = 0.05). This very low detrusor pressure (P_det_) was significantly greater than zero (p = 0.03).

### Changes in hypogastric nerve activity during bladder filling

#### In intact bladders

**Fig 5:**
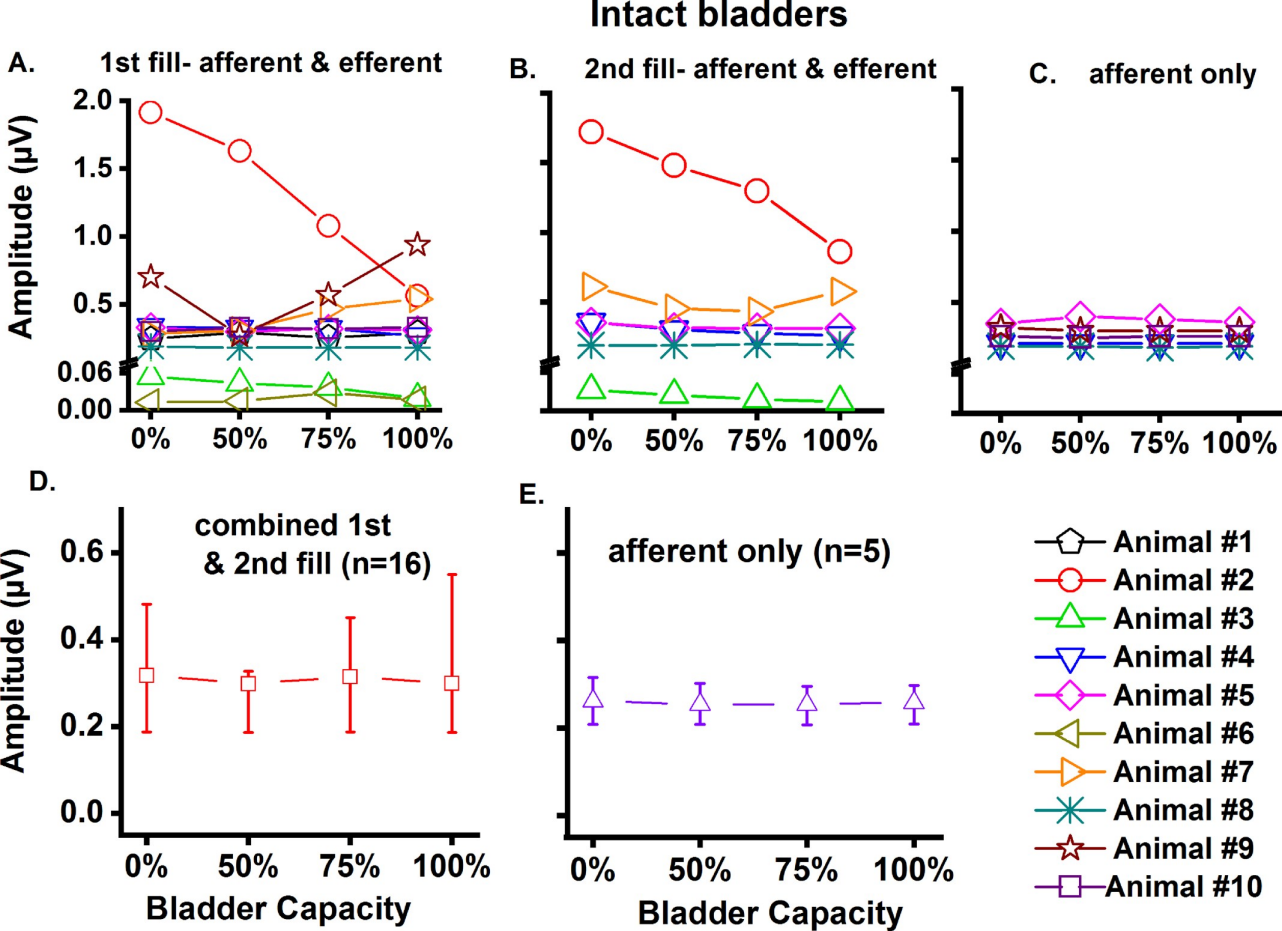
Hypogastric nerve recording during bladder filling in intact bladders. A) First fill (n = 10 animals); B) second fill (n = 6 animals); C) during bladder filling after elimination of efferent inputs (n = 5 animals). Panels A-C: data obtained for individual animals. Panels D-E: median & interquartile range (25%, 75%) was calculated.

We next examined the effects of bladder filling on hypogastric nerve recordings in 10 animals with intact bladders (Fig 5). In animals #1, #4, #5, #8 and #10, nerve activity remained unchanged during the 1st fillings, and during the 2nd bladder filling in animals #4, #5 and #8 (Fig 5A and 5B). However, in animals #2, and #3, we observed consistent decreases in amplitude with increasing bladder pressure (P_ves_) during both fillings (Fig 5A and 5B). In contrast, increases were seen in animals #6, #7 and #9, although at different bladder capacities. Specifically, animal #6 showed an increased amplitude between 50% and 75% capacity, yet a decrease between 75%-100% during the 1st filling (a 2nd filling was not performed). In animal #7, amplitude increased progressively with increased bladder filling during the 1st filling (Fig 5A). During the 2nd filling, decreased amplitudes was observed at 50% and 75%, followed by an increase at 100%. In #9, the amplitude decreased at 50% capacity and increased at 100% (a 2nd filling was not performed).

Next, only afferent activity was monitored during bladder filling after proximal hypogastric nerve transection (n = 5, Fig 5C). In animal #5, the amplitude slightly increased initially at 50% capacity and then decreased as capacity reached 75%-100%. No change in afferent nerve activity was observed during filling in the remaining animals.

When individual recording results were combined (because no difference was found in the amplitude during 1st and 2nd filling, we combined these), no statistically significant difference was found in nerve activity at 100% capacity (Fig 5D). After elimination of efferent inputs, no statistically significant difference was found in the afferent activity (Fig 5E).

#### In acutely lumbosacral decentralized bladders

Two of 3 recordings performed to assay background noise showed no change during bladder filling. In one recording, amplitude increased during filling and a blood clot was found inside the electrode.

**Fig 6:**
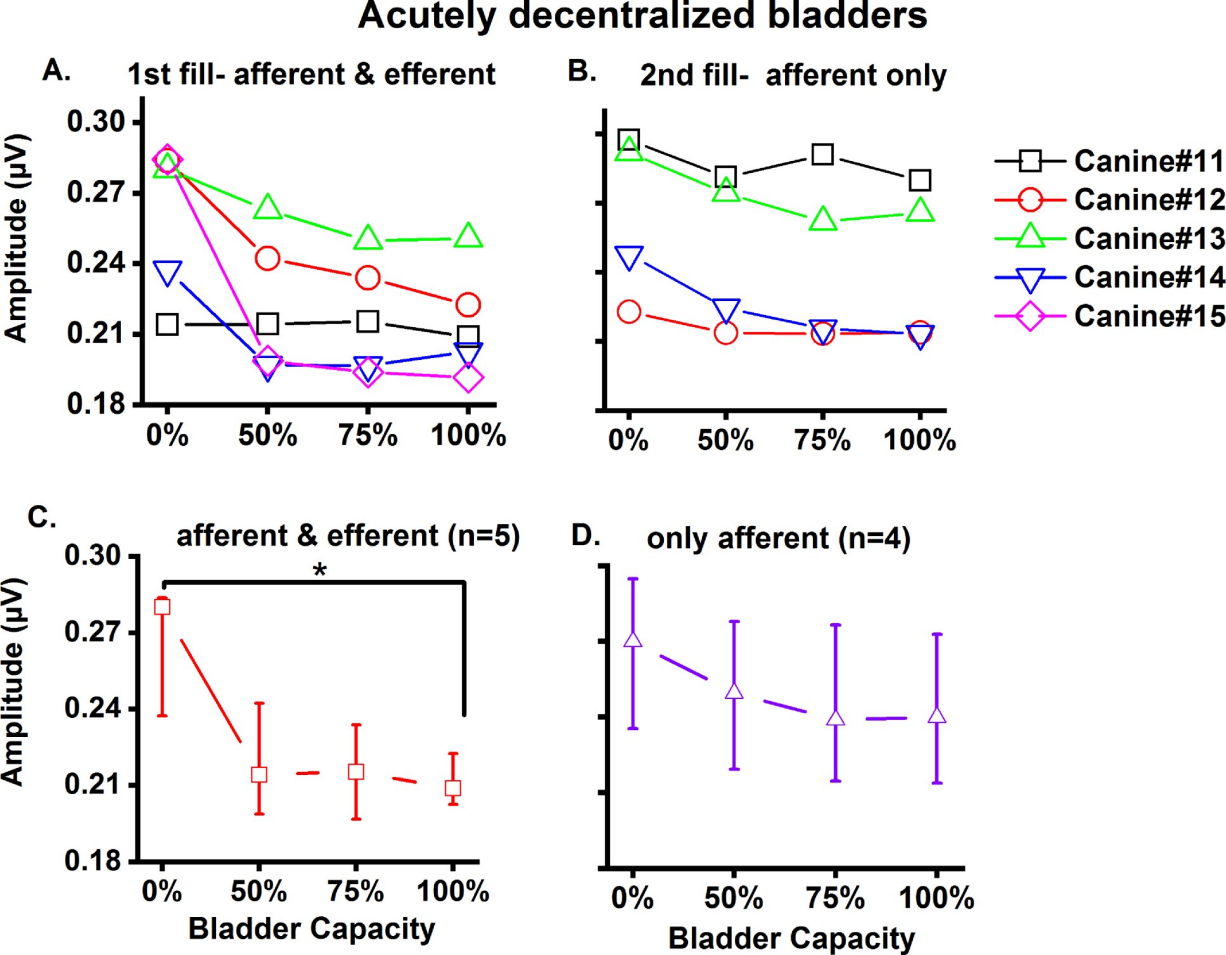
Hypogastric nerve recording in acutely decentralized (L6-L7 dorsal roots and all roots caudal to L7) bladders. A) 1st fill (n = 5 animals); B) 2nd fill after elimination of efferent inputs (n = 4 animals). Panels A-B: data obtained for individual animals. Panels C-D: median & interquartile range (25%, 75%) was calculated (*p = 0.03).

We next examined the results of hypogastric nerve activity (both efferent and afferent activity) individually during 1st filling (n = 5, Fig 6A). In animal #11, there was no change in amplitude when pressure (P_ves_) reached 75% capacity and then only a slight decrease was observed at 100% capacity. In animals #12, #13, #14, and #15, decreased activity was observed at 50% capacity. In #14, activity increased slightly thereafter, while #12, #13 and #14 showed decreases.

Afferent nerve discharges were also recorded during 2nd filling (n = 4, Fig 6B). In #11, activity decreased from 0%-50% capacity, increased transiently from 50%-75% capacity, before decreasing again as the bladder reached 100% capacity. The remaining (#12, 13 and 14) showed consistent decreases when the bladder reached 75% capacity and remained unchanged or slightly increased at 100% capacity. When these results were combined, nerve activity decreased statistically significantly at 100% capacity during the 1st filling (Fig 6C).

After elimination of efferent inputs, no statistically significant difference was found at 100% capacity (Fig 6D). We observed that afferent activity at 0% capacity was higher as compared to both afferent and efferent activity (Fig 6C and 6D). This is attributed to the nerve activity not completely settling to baseline prior to the subsequent recording.

#### Nerve histology

Positive tyrosine hydroxylase immunostaining confirmed the collected nerves as sympathetic nerves (hypogastric) in all 5 animals (S4 Fig).

## Discussion

Although it has been suggested that the type of anesthetic used can dose-dependently hinder detection of sensory or motor evoked potentials [25, 27], we found no differences between isoflurane versus propofol on detrusor contraction in response to hypogastric nerve stimulation.

We next determined the effect of hypogastric nerve stimulation on detrusor pressure in intact bladders. Previous studies by de Groat and colleagues in cats [28] and Elmer in rats [29] showed that electrical stimulation of hypogastric nerves (with intact bladder innervation) elicited low-amplitude bladder contractions. Our results in mongrel hound dogs show similarly that electrical stimulation of hypogastric nerves with intact bladders evokes low amplitude detrusor contractions. These contractions, although statistically significantly greater than zero, may not be physiologically sufficient to overcome outlet resistance for bladder emptying as compared to the S1-S3 cord/root stimulations that evoked large amplitude contractions up to 25 cm H_2_O.

We examined the effect of hypogastric nerve stimulation induced detrusor contraction in different degrees of acute spinal root transection as compared to intact bladders. Previously, a study by de Groat in cats confirmed that the response evoked by stimulation of the hypogastric nerve at 2 weeks after sacral root transection was similar to evoked bladder responses from the intact side, and that these responses were influenced by the length of the recovery period following sacral root transection [28]. Our results confirmed in mongrel hound dogs that there was no change in hypogastric nerve stimulation evoked contractions after spinal root transection at different segment levels as compared to intact bladders.

The response of lumbar (L1-L7) stimulation evoked detrusor pressure was determined with the hypogastric nerve intact, and showed that lower lumbar stimulation (L5-L7) minimally contributed to evoked pressure. Yet, L2 ventral root stimulation evoked strong contractions that remained unchanged after transection of L6 & L7 dorsal and all roots caudal to L7 with hypogastric nerves left intact, compared to intact bladders. However, transection of hypogastric nerves and all roots caudal to L5 resulted in significantly decreased, although not absent, L2 stimulation-evoked detrusor contraction.

Our results confirm prior findings in cats and rats that upper lumbar preganglionic axons pass through the caudal mesenteric ganglia and the hypogastric nerve to the pelvic plexus and bladder [30, 31]. Also, we have reported direct projections from ventral horn regions of the lumbosacral spinal cord to the bladder using retrograde dye labeling [18, 19, 21, 32]. Based on these findings, the residual, low amplitude evoked contraction during L2 spinal root stimulation after transection of hypogastric nerves and all roots caudal to L5 is likely due to low numbers of direct projections from the L2 ventral horn to the bladder. This is the first study showing functional motor effects of these direct upper lumbar ventral horn projections to the bladder that, while statistically significantly detectable, may be so low as to not be physiologically significant for bladder emptying.

Lastly, we wanted to determine the extent of afferent activity from the bladder present in the hypogastric nerve that might be contributing to bladder sensory function. Previous data from cats indicate that hypogastric nerve activity remains silent during initial bladder filling and increases 21% by the time the bladder reaches half of its capacity [33]. In our recordings with intact bladders, although the afferent and efferent activity in individual animals showed variability, we did not find any change in combined nerve activity at different bladder capacities. After bilateral transection of L6 & L7 dorsal roots and all roots caudal to L7, total hypogastric nerve activity decreased during filling. The reason for the decrease in nerve activity is unknown. Our observation that afferent activity did not change in both intact and decentralized bladders during filling might suggest that afferent fibers in the hypogastric nerve mostly remain silent during bladder filling. Base line amplitude of raw ENG in individual dogs varied from 2 μV-5 μV that results low SNR in recorded activity in some animals. Although, recorded nerve signals have very low SNR and inconsistency during repetitive fillings, our measurement of changes in nerve activity is encouraging. This is the first study reporting assessment of hypogastric afferent and efferent activity during bladder storage in a canine model.

## Conclusions

In this study, we stimulated hypogastric nerve and lumbo-sacral cord/roots. We conclude that a more complete decentralized canine bladder model requires transection of the lumbosacral spinal roots innervating the bladder as well as the hypogastric nerve prior to reinnervating the bladder with nerve transfer surgeries. We also found functional motor effects of upper lumbar ventral horn projections to the bladder that may not be physiologically significant for bladder emptying.

We designed custom cuff electrodes and improved upon electrophysiological methods, with emphasis on ENG recordings. We applied these refined methods in electrophysiological experiments and recorded hypogastric nerve firing activity. We plan to implement these refined techniques in a larger study assessing sensory and motor reinnervation of the bladder following lower motor neuron lesion and bladder reinnervation with nerve transfer surgery.

## Supporting information

S1 Table Results of electrodes testing. (DOCX)

S1 Fig Step-by-step testing of A/D converter and amplifier to calibrate noise in saline. A) Grounded source input channel A/D Converter and measured amplitude of output noise. B) Grounded all channels of A/D converter and measured amplitude of output noise. C) Connected differential amplifier output to an input of A/D converter with amplifier’s inputs shorted together to measure internal noise produced by instrumentation. D) In-vitro saline setup for recording. ENG: Electroneurogram.
(TIF)

S2 Fig Study design. Hypogastric nerve stimulation and recording were performed under different surgical conditions. Lumbosacral spinal cord/roots stimulations were performed in intact bladders, followed by stimulation of L2 ventral roots before and after hypogastric nerve transection under different surgical conditions.
(TIF)

S3 Fig Example of hypogastric nerve stimulation and recording. A) Maximum detrusor pressure was recorded at 10/s sampling rate during nerve stimulation (period 49 of stimulation indicated by “on” and “off”): 10 data sample (in 1 second time window) were taken to calculate the mean peak and baseline detrusor pressures. B) Hypogastric nerve recording during bladder filling were performed at 20k/s sampling rate. Top trace: bladder pressure; Middle trace: ENG data (500 Hz-3 kHz); bottom trace: Root mean square (RMS) of the amplitude of raw ENG data within a 10s window.
(TIF)

S4 Fig Hypogastric nerve sections stained for tyrosine hydroxylase using immunohistochemical methods. A and B) Two different examples of hypogastric nerves probed with specific antibodies to tyrosine hydroxylase. Images taken with a 40x microscope objective.
(TIF)

S5 Fig Amplitude of base line noise measured in saline. A) AC line operated amplifier, and B) Battery operated amplifier. Battery operated amplifier showed 80% reduction in base line noise amplitude, compared to an AC line operated amplifier.
(TIF)
