# Supplementary material for: Acute bladder decentralization in hound dogs: Preliminary results of effects on hypogastric nerve electroneurograms and detrusor pressure responses to spinal root and hypogastric nerve stimulation
Source: PLoS One. 2019 Apr 10;14(4):e0215036. doi: 10.1371/journal.pone.0215036 (PMC6457673; doi:10.1371/journal.pone.0215036)
Supplement: S1 Table — (DOCX) [file pone.0215036.s001.docx]

# **Supporting information**

# **Supplemental methods**

## Electrodes design

The continuity test was performed to ensure that there was no breakage in lead wires. The bubble test to determine connectivity of the electrode electrical leads was performed in 0.9% NaCl (saline) using a 6 volt DC battery. When the battery was connected, we examined for a visible stream of bubbles from the electrode surfaces. Last, an impedance test was performed in saline.

## Supplemental Results

### **Functionality of bipolar and tripolar electrodes**

Functionality of designed electrodes was tested using several different assays (S1 Table). Continuity testing showed that electrodes lead wire resistances varied slightly relative to their inner diameters, which was considered negligible. All electrodes passed the bubble test showing proper functioning with no breaks or short circuits. The impedance of all electrodes ranged between 1-3 kΩ in saline solution and appeared to depend on the inner diameter of the electrodes (primarily in the bipolar configuration).

**S1 Table. Results of electrodes testing**

| **Electrode type** | **Inner diameter (millimeter)** | **Continuity test (Ω)** | **Bubble test** | **Impedance test (kΩ)** |
| --- | --- | --- | --- | --- |
| Tripolar | 3.0 | 33 | Pass | 1.0 |
| Tripolar | 2.4 | 45 | Pass | 1.5 |
| Tripolar | 1.7 | 41 | Pass | 1.0 |
| Tripolar | 1.5 | 42 | Pass | 1.5 |
| Tripolar | 1.2 | 46 | Pass | 1.0 |
| Bipolar | 3.0 | 44 | Pass | 1.0 |
| Bipolar | 1.7 | 44 | Pass | 2.0 |
| Bipolar | 0.8 | 64 | Pass | 3.0 |

### **Calibrated noise in saline**

With the recording setup shown in S 1 Fig., noise in the range of ± 10 μV peak-to-peak was found when using the AC power line operated differential amplifier Model 1700 (S5 Fig.). When the battery operated low noise voltage preamplifier Model SR560 was used, a noise range between ±2 μV_peak-to-peak_ was observed, an 80% reduction (S5 Fig.).

**S1 Fig. Step-by-step testing of A/D converter and amplifier to calibrate noise in saline.** A) Grounded source input channel A/D Converter and measured amplitude of output noise. B) Grounded all channels of A/D converter and measured amplitude of output noise. C) Connected differential amplifier output to an input of A/D converter with amplifier’s inputs shorted together to measure internal noise produced by instrumentation. D) In-vitro saline setup for recording. ENG: Electroneurogram

**S2 Fig. Study design.** Hypogastric nerve stimulation and recording were performed under different surgical conditions. Lumbosacral spinal cord/roots stimulations were performed in intact bladders, followed by stimulation of L2 ventral roots before and after hypogastric nerve transection under different surgical conditions.

**S3 Fig. Example of hypogastric nerve stimulation and recording.** A) Maximum detrusor pressure was recorded at 10/s sampling rate during nerve stimulation (period of stimulation indicated by “on” and “off”): 10 data sample (in 1 second time window) were taken to calculate the mean peak and baseline detrusor pressures. B) Hypogastric nerve recording during bladder filling were performed at 20k/s sampling rate. Top trace: bladder pressure; Middle trace: ENG data (500 Hz-3 kHz); bottom trace: Root mean square (RMS) of the amplitude of raw ENG data within a 10s window.

**S4 Fig. Hypogastric nerve sections stained for tyrosine hydroxylase using immunohistochemical methods.** A and B) Two different examples of hypogastric nerves probed with specific antibodies to tyrosine hydroxylase. Images taken with a 40x microscope objective.

**S5 Fig. Amplitude of base line noise measured in saline.** A) AC line operated amplifier, and B) Battery operated amplifier. Battery operated amplifier showed 80% reduction in base line noise amplitude, compared to an AC line operated amplifier.

## Supplemental Conclusions

## Electrode testing results showed similar outputs for different electrode configurations allowing use of either configuration for recordings. We preferred the bipolar configuration due to limited implantation sites (S2 Table). Testing results from AC line amplifier versus battery operated amplifier in saline solutions, confirmed the lower noise of the battery-operated amplifier for further recordings (S5 Fig.).
