# Supplementary figures and images for: Acute bladder decentralization in hound dogs: Preliminary results of effects on hypogastric nerve electroneurograms and detrusor pressure responses to spinal root and hypogastric nerve stimulation
Source: PLoS One. 2019 Apr 10;14(4):e0215036. doi: 10.1371/journal.pone.0215036 (PMC6457673; doi:10.1371/journal.pone.0215036)

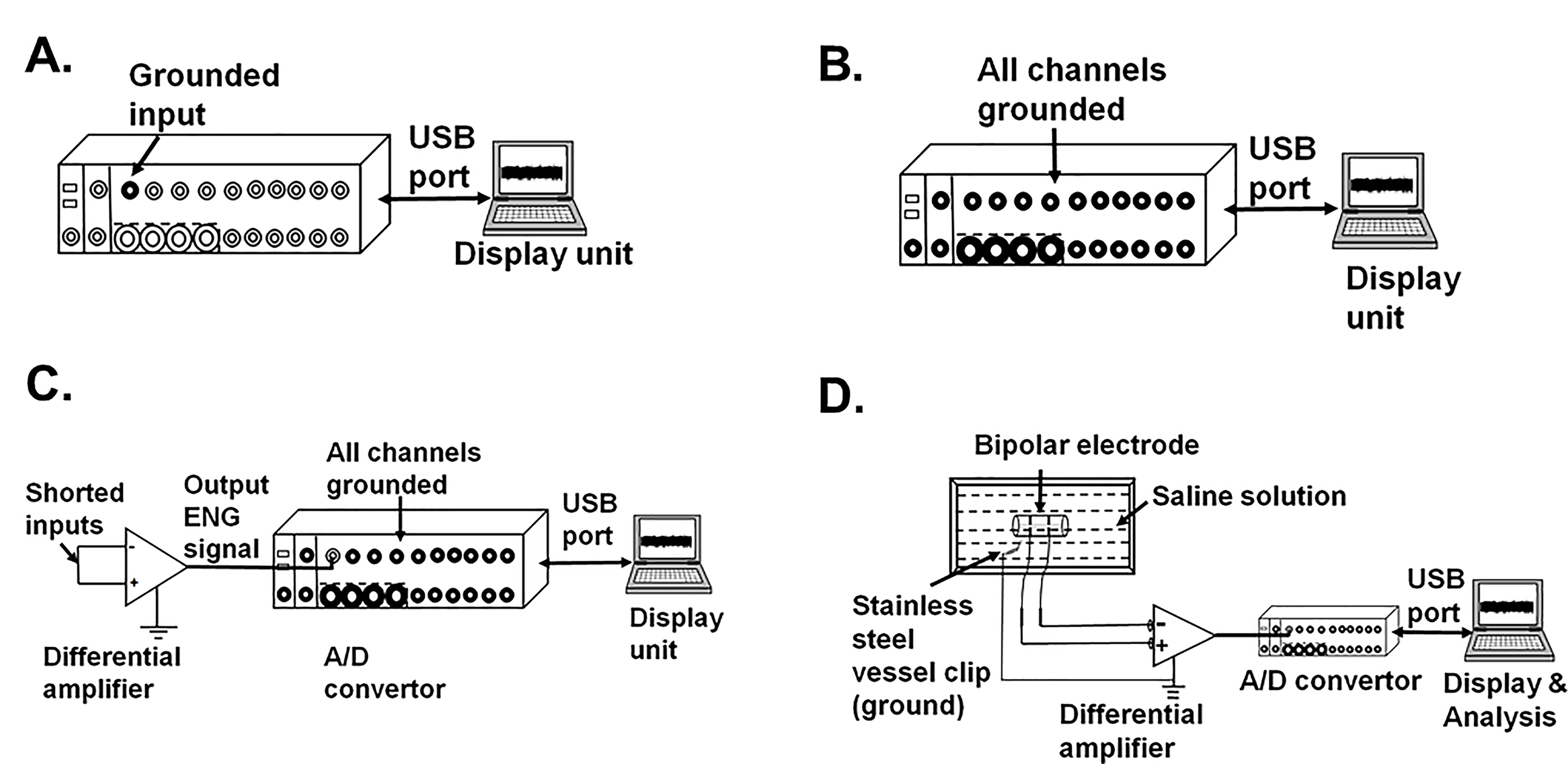

Supplement: S1 Fig — A) Grounded source input channel A/D Converter and measured amplitude of output noise. B) Grounded all channels of A/D converter and measured amplitude of output noise. C) Connected differential amplifier output to an input of A/D converter with amplifier’s inputs shorted together to measure internal noise produced by instrumentation. D) In-vitro saline setup for recording. ENG: Electroneurogram. (TIF) [file pone.0215036.s002.tif]

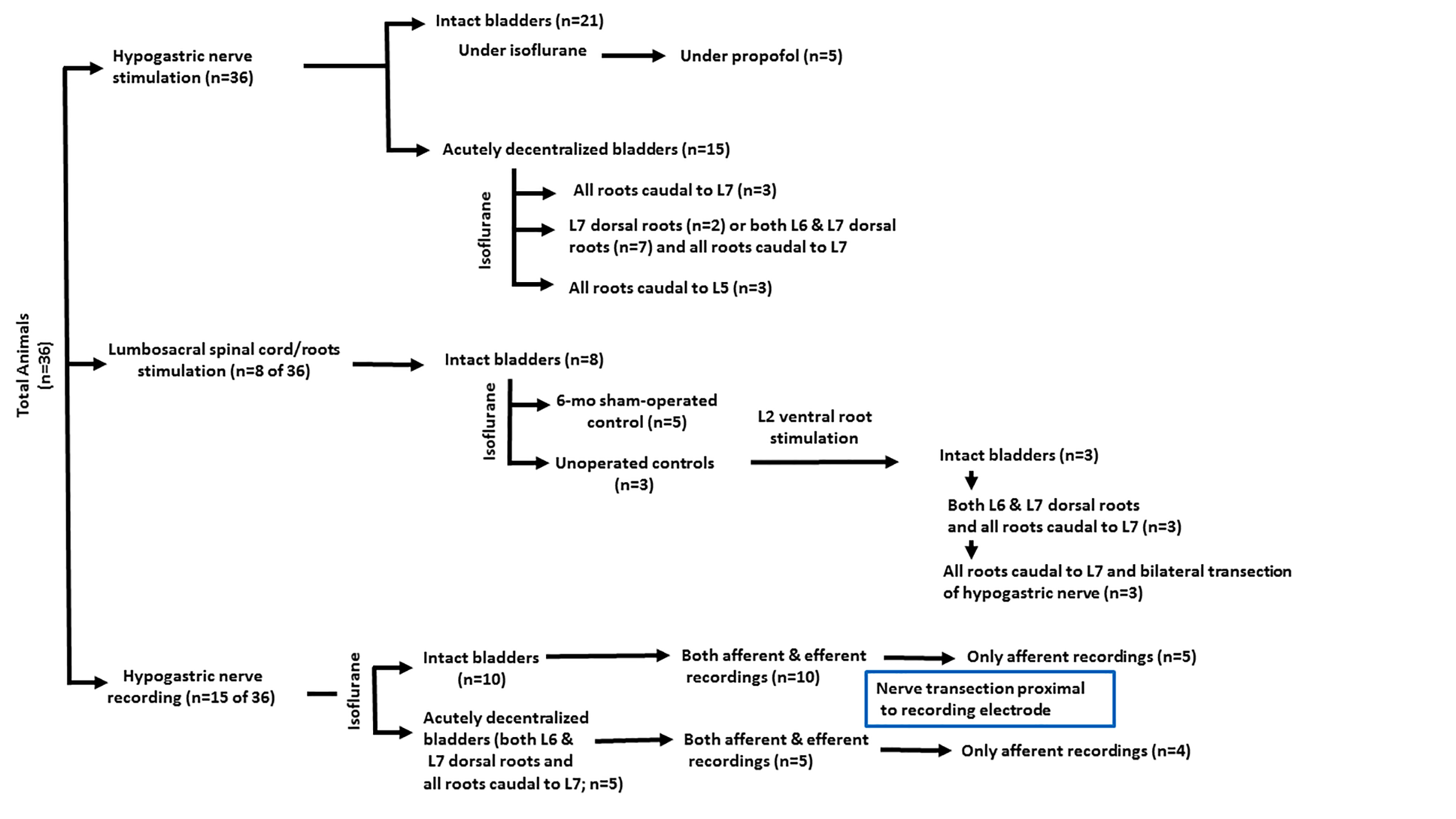

Supplement: S2 Fig — Hypogastric nerve stimulation and recording were performed under different surgical conditions. Lumbosacral spinal cord/roots stimulations were performed in intact bladders, followed by stimulation of L2 ventral roots before and after hypogastric nerve transection under different surgical conditions. (TIF) [file pone.0215036.s003.tif]

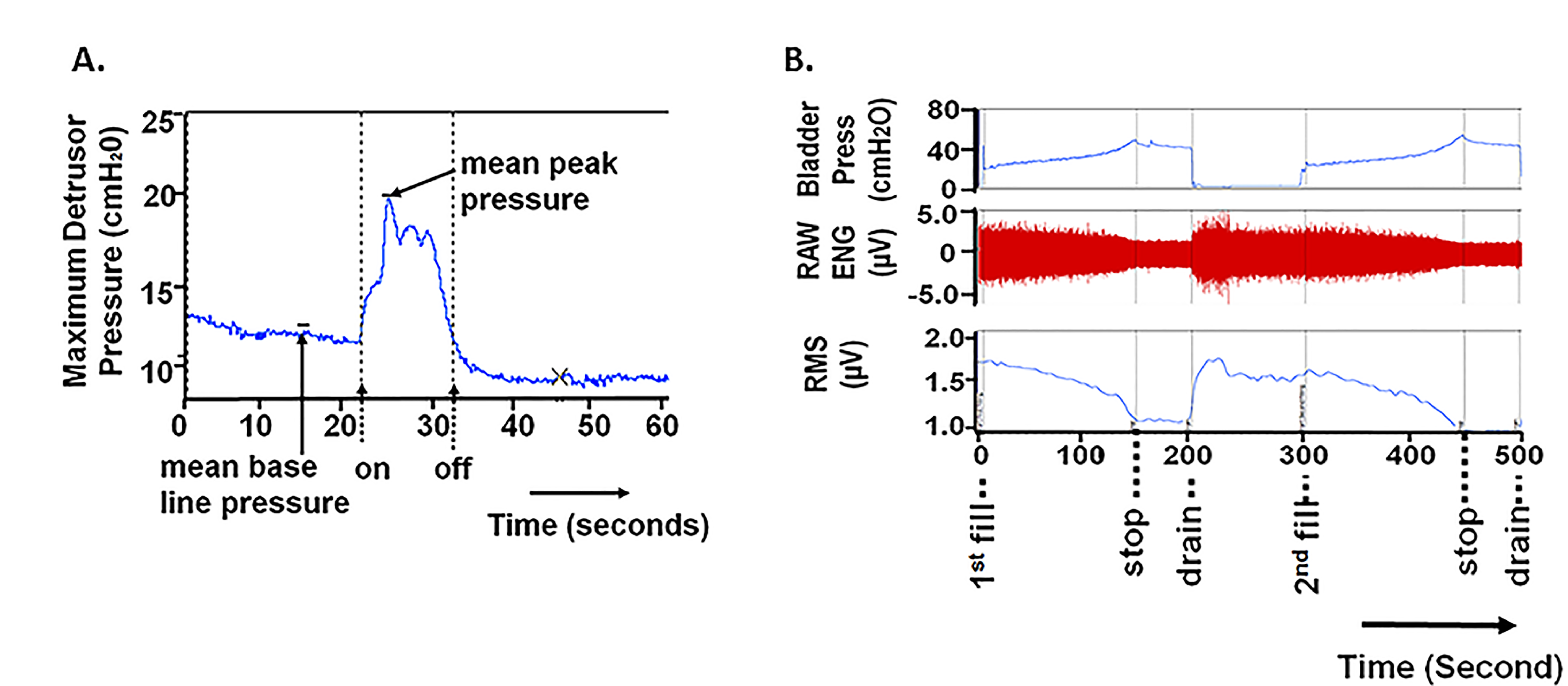

Supplement: S3 Fig — A) Maximum detrusor pressure was recorded at 10/s sampling rate during nerve stimulation (period 49 of stimulation indicated by “on” and “off”): 10 data sample (in 1 second time window) were taken to calculate the mean peak and baseline detrusor pressures. B) Hypogastric nerve recording during bladder filling were performed at 20k/s sampling rate. Top trace: bladder pressure; Middle trace: ENG data (500 Hz-3 kHz); bottom trace: Root mean square (RMS) of the amplitude of raw ENG data within a 10s window. (TIF) [file pone.0215036.s004.tif]

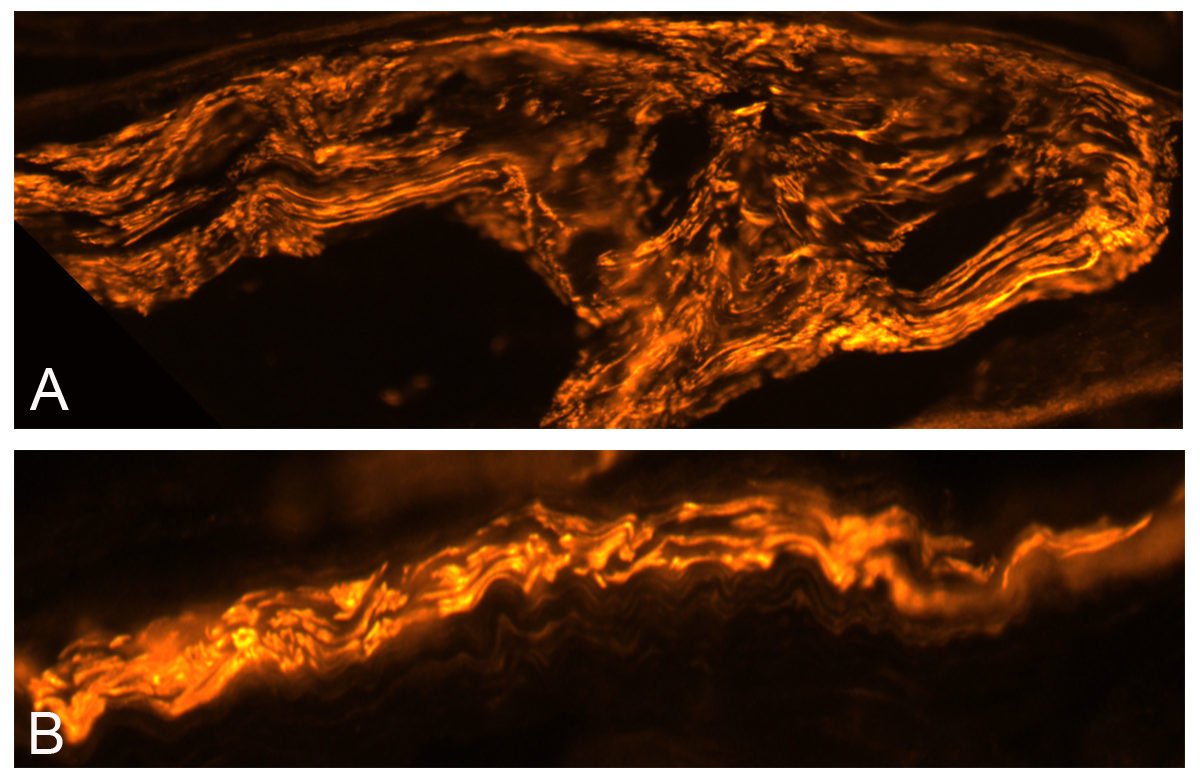

Supplement: S4 Fig — A and B) Two different examples of hypogastric nerves probed with specific antibodies to tyrosine hydroxylase. Images taken with a 40x microscope objective. (TIF) [file pone.0215036.s005.tif]

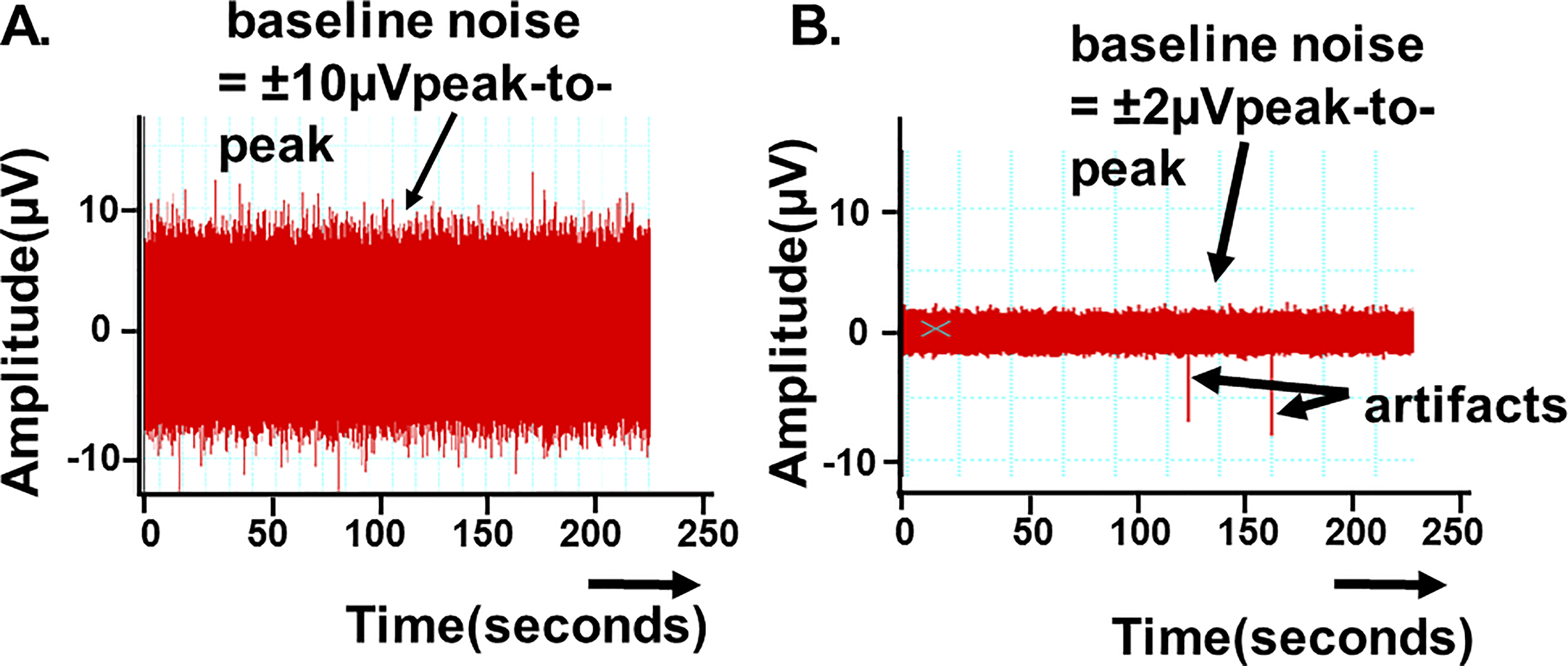

Supplement: S5 Fig — A) AC line operated amplifier, and B) Battery operated amplifier. Battery operated amplifier showed 80% reduction in base line noise amplitude, compared to an AC line operated amplifier. (TIF) [file pone.0215036.s006.tif]
